# Anterior Cruciate Ligament Reconstruction Using Lateral Extra-Articular Procedures: A Systematic Review

**DOI:** 10.3390/medicina61020294

**Published:** 2025-02-08

**Authors:** Filippo Migliorini, Ludovico Lucenti, Ying Ren Mok, Tommaso Bardazzi, Riccardo D’Ambrosi, Angelo De Carli, Domenico Paolicelli, Nicola Maffulli

**Affiliations:** 1 Department of Orthopaedic and Trauma Surgery, Academic Hospital of Bolzano (SABES-ASDAA), 39100 Bolzano, Italy; tommaso.bardazzi@sabes.it; 2 Department of Life Sciences, Health, and Health Professions, Link Campus University, 00165 Rome, Italy; 3 Department of Precision Medicine in Medical, Surgical and Critical Care (Me.Pre.C.C.), University of Palermo, 90133 Palermo, Italy; ludovico.lucenti@gmail.com; 4 Division of Sports Shoulder and Elbow Surgery, Department of Orthopaedic Surgery, National University Hospital, Singapore 119074, Singapore; ying_ren_mok@nuhs.edu.sg; 5 IRCCS Galeazzi Hospital, Sant’Ambrogio, 20157 Milan, Italy; riccardo.dambrosi@hotmail.it; 6 Dipartimento di Scienze Biomediche per la Salute, University of Milan, 20122 Milan, Italy; 7 Department of Trauma and Orthopaedic Surgery, Faculty of Medicine and Psychology, University La Sapienza, 00185 Rome, Italyn.maffulli@qmul.ac.uk (N.M.); 8 School of Pharmacy and Bioengineering, Keele University Faculty of Medicine, Stoke on Trent ST4 7QB, UK; 9 Centre for Sports and Exercise Medicine, Barts and the London School of Medicine and Dentistry, Mile End Hospital, Queen Mary University of London, London E1 4DG, UK

**Keywords:** anterior cruciate ligament, lateral extraarticular tenodesis, PROMs, return to sport, complications

## Abstract

*Background and Objectives*: The present systematic review investigated the efficacy of lateral extra-articular tenodesis (LET) and anterolateral ligament (ALL) as lateral extra-articular procedures (LEAPs) for anterior cruciate ligament (ACL) reconstruction. ACL reconstruction using LEAP may reduce graft rupture and rotatory laxity and allow a quicker return to sports. The outcomes of interest were patient-reported outcome measures (PROMs), return to sport, laxity, failure rate, and safety profile. *Materials and Methods*: The present systematic review followed the 2020 PRISMA guidelines. In December 2024, PubMed, EMBASE, and Web of Science were accessed without constraints. All clinical investigations evaluating LEAP for ACL reconstruction were considered. Only studies that considered LET and ALL as LEAP were considered. Only studies using a hamstring tendon autograft associated with LET or ALL were considered. *Results*: Data from 27 clinical studies (3423 patients) were retrieved. The mean length of follow-up was 61.8 ± 39.5 months. ACL reconstruction using LEAP led to a statistically significant improvement in the Lysholm score (*p* < 0.01) and IKDC (*p* < 0.01). The mean joint laxity, as measured by the arthrometer, was 1.5 ± 1.8 mm. Finally, 72.3% (623 of 668) of patients returned to their pre-injury level of sport at a mean of 6.3 ± 4.4 months. At the last follow-up, the LET group showed greater IKDC (*p* = 0.04). On the other hand, there was a statistically significant greater rate of patients positive to the Lachman test (*p* < 0.01), return to sport (*p* < 0.01), and reoperation (*p* = 0.01). No significant differences were found in Lysholm scores (*p* = 0.6), Tegner scores (*p* = 0.2), arthrometer measurements (*p* = 0.2), Pivot shift test results (*p* = 0.1), time to return to sport (*p* = 0.3), and failure rates (*p* = 0.7). *Conclusions*: LEAP for ACL reconstructions seems to be effective and safe. Most patients returned to their pre-injury level of sport after a mean of 6 months. LET-based ACL reconstruction may be associated with greater clinical outcomes and a higher reoperation rate compared to ALL-based reconstruction.

## 1. Introduction

The prevalence of anterior cruciate ligament (ACL) injuries among young athletes has risen, with an annual increase of approximately 2.3% over the past two decades [1,2,3,4,5,6]. This trend is primarily attributed to the increasing involvement of youth in high-intensity competitive sports [7,8]. While traditional single-bundle ACL reconstruction has shown promise, concerns persist regarding residual rotational instability [9,10,11,12,13,14]. Previous studies have indicated that residual rotational instability persists in up to 25% of patients, often leading to suboptimal outcomes [15,16,17]. Researchers have explored various techniques to enhance stability, including anatomic and double-bundle reconstructions [18,19,20]. More recently, attention has shifted towards lateral extra-articular procedures (LEAP) for ACL reconstruction, specifically anterolateral ligament (ALL) reconstruction and lateral extra-articular tenodesis (LET) [21,22,23,24,25,26,27,28]. ACL reconstruction using LEAP may reduce graft rupture and rotatory laxity and allow a quicker return to sport [29,30]. The optimal surgical method is still debated, and previous investigations have mainly focused on PROMs, knee laxity, return to sport, and safety [31,32,33,34,35]. Initially introduced by Lemaire [36], LET has demonstrated improved anterolateral stability and clinical outcomes when combined with ACL reconstruction in several randomised controlled trials [25,29,33,37,38,39,40,41,42,43]. ALL has also emerged as an alternative for addressing rotational instability [2,24,40,44,45,46,47,48,49,50]. Given the advancements in understanding the anterolateral ligament complex, ALL offers a potentially more anatomically accurate reconstruction [47,51,52,53,54]. Despite these developments, evidence on clinical outcomes between LET and ALL in ACL reconstruction is still limited, and the optimal technique is still under debate. Previous biomechanical studies using cadaveric models have suggested superior properties for LET compared to ALL [55,56]. This systematic review aims to bridge this knowledge gap by evaluating the outcomes of LEAP and comparing outcomes of ALL and LET in ACL reconstruction regarding patient-reported outcome measures (PROMs), return to sport, joint laxity, and complication rate. The endpoint choice relates to their common use and recognised validity in clinical practice and research [57,58].

## 2. Methods

### 2.1. Eligibility Criteria

All clinical investigations evaluating LEAP for ACL reconstruction were considered. Only studies that considered LET and ALL as LEAP procedures were considered. Only studies using a hamstring tendon autograft associated with LET or ALL were considered. According to the authors’ capabilities, only articles published in the following languages were included: English, Italian, German, French, and Spanish. Eligible studies had to be published in peer-reviewed journals. Only studies with levels I to III of evidence, according to the Oxford Centre of Evidence-Based Medicine (OCEBM), [59] were included. Opinions, editorials, letters, and reviews were excluded, as were studies involving computational analyses, in vitro or animal experiments, biomechanical assessments, or cadaveric research. Studies focusing on allografts or synthetic grafts were also deemed ineligible. Finally, only studies with a minimum follow-up period of six months were considered, as this is the established cut-off for allowing sports resumption [60,61]. A shorter follow-up period could impair the assessment of the outcome [60,61].

### 2.2. Search Strategy

The present systematic review followed the guidelines defined in the 2020 Preferred Reporting Items for Systematic Reviews and Meta-Analyses (PRISMA) statement [62]. The following PICOT framework was established:

- P (Problem): ACL reconstruction;
- I (Intervention): LEAP;
- C (Comparison): LET vs. ALL;
- O (Outcomes): PROMs, complications, return to sport, laxity;
- T (Timing): minimum six months of follow-up.

### 2.3. Data Source

EMBASE, PubMed, and Web of Science were accessed in December 2024 without additional filters or temporal constraints. The Medical Subject Headings (MeSH) used in the database search were as follows: (“Anterior Cruciate Ligament”[Mesh] OR “Anterior Cruciate Ligament/physiopathology”[Mesh] OR “Anterior Cruciate Ligament Injuries”[Mesh] OR “Arthralgia/etiology”[Mesh] OR “Athletes”[Mesh] OR “Joint Instability/etiology”[Mesh] OR “Knee Joint”[Mesh] OR “Knee Joint / pathology”[Mesh] OR “Ligaments, Articular/injuries”[Mesh] OR anterior cruciate ligament OR Knee OR rotational instability OR Rotational laxity OR Knee injuries OR ACL OR anterior cruciate injuries OR ACL injuries) AND (“Anterior Cruciate Ligament/surgery”[Mesh] OR “Anterior Cruciate Ligament Injuries/surgery”[Mesh] OR “Anterior Cruciate Ligament Reconstruction”[Mesh] OR “Hamstring Tendons/surgery”[Mesh] OR “Hamstring Tendons/transplantation”[Mesh] OR “Joint Instability/surgery”[Mesh] OR “Knee Injuries/surgery”[Mesh] OR “Knee Joint/surgery”[Mesh] OR “Ligaments, Articular/surgery”[Mesh] OR “Tendon Transfer”[Mesh] OR “Tendons/transplantation”[Mesh] OR “Transplantation, Autologous”[Mesh] OR ACL reconstruction OR anterior cruciate ligament reconstruction OR Hamstring OR hamstring tendon graft OR reconstruction) AND (“Suture Techniques”[Mesh] OR “Tenodesis”[Mesh] OR “Tenodesis/methods”[Mesh] OR Anterolateral complex OR Anterolateral instability OR Anterolateral ligament OR Anterolateral ligament reconstruction OR Anterolateral reconstruction OR Combined with extraarticular tenodesis OR extra-articular reconstruction OR extra-capsular augmentation OR Lateral extra articular tenodesis OR Lateral tenodesis OR Lemaire procedure OR LET OR Modified Lemaire OR tenodesis OR ALL) AND (“Lysholm Knee Score”[Mesh] OR “Patient Reported Outcome Measures”[Mesh] OR “Postoperative Complications”[Mesh] OR “Quality of Life”[Mesh] OR “Range of Motion, Articular”[Mesh] OR “Recovery of Function”[Mesh] OR “Reoperation”[Mesh] OR “Return to Sport”[Mesh] OR “Treatment Outcome”[Mesh] OR graft failure OR graft rupture OR Pivot shift test OR Pivot-shift OR return to sport OR rotational stability OR PROMS OR Patient reported outcomes OR Lachman test).

### 2.4. Outcomes of Interest

Two authors (D.P. and T.B.) independently conducted data extraction. For each study, the following generalities were collected: author, year and journal of publication, study design, and length of the follow-up (months). The following data at baseline were retrieved: number of patients, number of women, mean age, and mean BMI. Data on the following PROMs at baseline and the last follow-up were retrieved: Lysholm score [58], International Knee Documentation Committee (IKDC) [63], and Tegner Activity Scale [58]. According to previously published reports, the minimally clinically important difference (MCID) is 13.8/100 for the IKDC, 9.9/100 for the Lysholm, and 0.5/10 for the Tegner Activity Scale [64,65,66,67]. Data on the KT-1000 and KT-2000 arthrometer [68], Pivot-shift, and Lachman tests were collected at the last follow-up to evaluate laxity. Data on the level of return and time to return to sport (months) were also collected. Data concerning failure (graft ruptures) and reoperation rates were extracted. Data extraction was performed using Microsoft Office Excel version 16.0 (Microsoft Corporation, Redmond, WA, USA).

### 2.5. Methodology Quality Assessment

Two authors (T.B. and D.P.) performed the methodological quality assessment. The revised Risk of Bias assessment tool (RoB2) [69,70], part of the Cochrane tool for assessing the Risk of Bias in randomised controlled trials (RoB) [71], was used. The following endpoints were considered: bias arising from the randomisation process, bias due to deviations from intended interventions, bias from missing outcome data, bias in the measurement of the outcome, and bias in the selection of reported results. Nonrandomised controlled trials (non-RCTs) were evaluated using the Risk of Bias in Nonrandomised Studies of Interventions (ROBINS-I) tool [72]. Seven domains of potential bias in non-RCTs were assessed. Two domains assess possible confounding factors and the nature of patient selection before the start of the comparative intervention. Bias in the classification of interventions is evaluated using another domain. The final four domains assess methodological quality after the intervention comparison has been implemented and relate to deviations from previously intended interventions, missing data, erroneous measurement of outcomes, and bias in the selection of reported outcomes. The figure of the ROBINS-I was elaborated using the Robvis Software (Risk-of-bias VISualization, Riskofbias.info, Bristol, UK) [73].

### 2.6. Statistical Analysis

The main author (F.M.) performed the statistical analyses following the recommendations of the Cochrane Handbook for Systematic Reviews of Interventions [74]. Descriptive statistics were calculated using IBM SPSS software, version 25 (International Business Machines Corporation, Armonk, NY, USA). Baseline comparability was assessed using the *t*-test, with *p* > 0.05 considered satisfactory. The arithmetic mean and standard deviation were used to report continuous data. The odd ratio (OR) was used as the effect measure for comparing dichotomous data. The unpaired *t*-test and the χ^2^ test were performed for continuous and dichotomic data, respectively, with values of *p* < 0.05 considered statistically significant.

## 3. Results

### 3.1. Search Result

**Figure 1:**
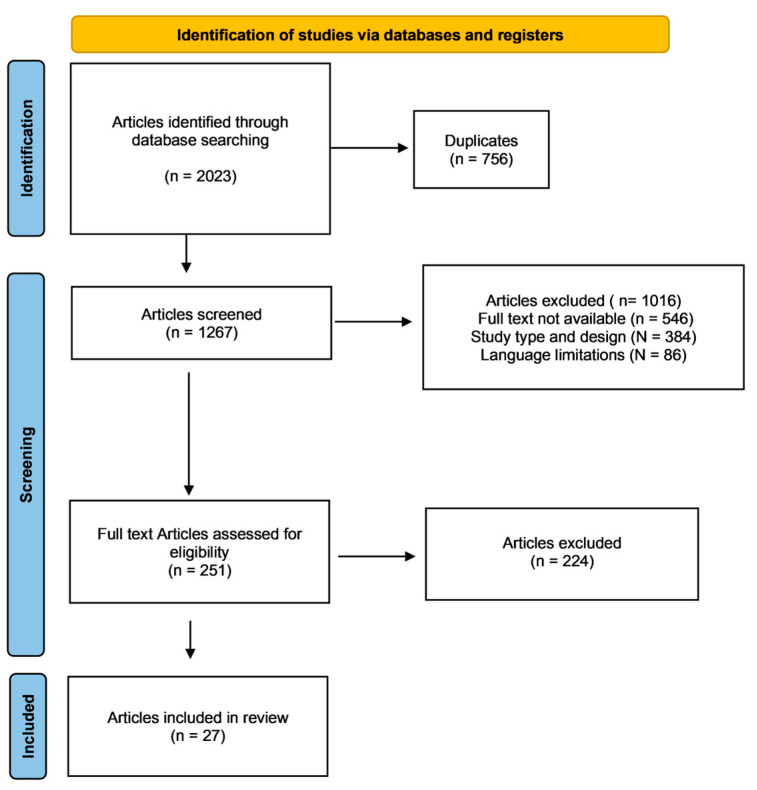
Flowchart of the literature search.

The systematic literature search resulted in the identification of 2023 articles. After removing duplicates, the abstracts of 1267 articles were screened for eligibility. A total of 1016 articles were excluded for the following reasons: mismatch with the predefined study design criteria (N = 384), full-text unavailability (N = 546), and language limitations (N = 86). Of the remaining 251 studies, another 224 were excluded after full-text evaluation. Consequently, a final selection of 27 studies was included in this systematic review. The literature search results are shown in Figure 1.

### 3.2. Methodological Quality Assessment

**Figure 2:**
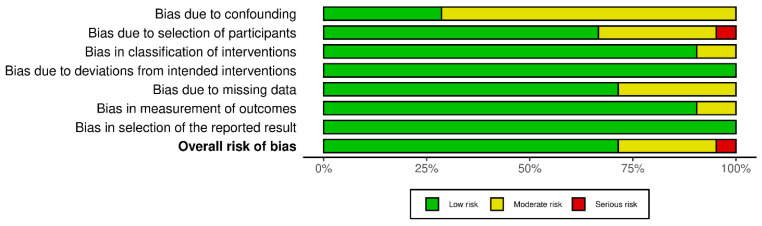
ROBINS-I of the non-RCTs.

The risk of bias in the 21 included non-RCTs was assessed using the ROBINS-I risk of bias. The risk of bias due to confounding was moderate in nearly three-fourths of the included non-RCTs. No bias was identified due to deviations from the intended intervention or the selection of reported results. Moreover, the risk of bias in all the other domains was judged to be low or moderate for nearly all studies. Only one article had significant issues in participant selection and was therefore classified as having a high risk of bias. Overall, 75% of the articles presented a low risk of bias, and the remaining non-RTS was found ut a moderate RoB, indicating an acceptable methodological quality (Figure 2).

**Figure 3:**
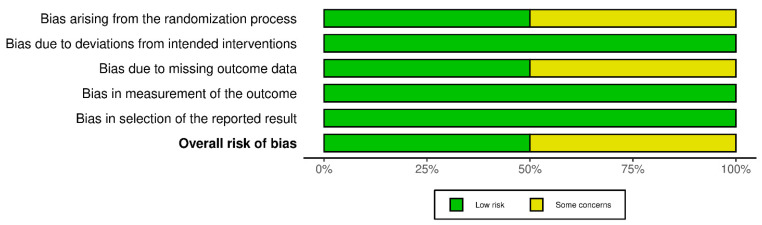
ROB2 of the RCTs.

The Cochrane risk of bias assessment tool (ROB 2) was used to evaluate the six included RCTs. The analysis suggested a low risk of bias for half the articles in the first and third domains, while a moderate risk emerged in the remaining articles. Al other domains presented no concerns for any of the included RCTs. The overall RoB was estimated to be low for three articles and moderate for the others, suggesting acceptable methodological quality. Figure 3 shows the bias risk distribution across the included RCTs.

### 3.3. Patient Demographics

Data from 3423 patients were retrieved, 34.2% of whom (767 of 2245) were women. The mean length of follow-up was 61.8 ± 39.5 months. The mean age was 25.1 ± 4.3 years, and the mean BMI was 23.6 ± 1.6 kg/m^2^. Table 1 shows the generalities and demographics of the study.

### 3.4. Baseline Comparability

Between the groups, baseline comparability was evidenced in the mean length of follow-up, mean age, mean BMI, female/male ratio, Lysholm score, and IKDC score (Table 2).

### 3.5. Efficacy of LEAP

ACL reconstruction using LEAP led to a statistically significant improvement in the Lysholm score (MD 31.4; *p* < 0.01) and IKDC (MD 29.1; *p* < 0.01) (Table 3). Good results were also found at the last follow-up in the Tegner score (6.8 ± 2.0); however, given the missing data at admission, its improvement was not analysed.

The Pivot shift and Lachman tests were positive in 15.8% (105 of 666) and 13.0% (87 of 668 patients), respectively. The mean joint laxity measured with the arthrometer was 1.5 ± 1.8 mm. Finally, 72.3% (623 of 668) of patients returned to the pre-injury level of sport at a mean of 6.3 ± 4.4 months. These data are shown in greater detail in Table 4.

### 3.6. Comparison of ALL Versus LET

At the last follow-up, the IKDC scored greater in the LET group (MD 5.6; *p* = 0.04). No differences were found in the Lysholm (*p* = 0.6) and Tegner (*p* = 0.2) scores. No difference was found in laxity measured with the arthrometer (*p* = 0.2) and in the Pivot shift test (*p* = 0.1). On the other hand, the ALL group evidenced a statistically significantly greater rate of patients testing positive for the Lachman test (OR 3.1; *p* <0.01). No difference was found in the time to return to sport (*p* = 0.3), while a statistically significant higher rate of return to sport was found in the LET group (OR 0.5; *p* < 0.01). These results are shown in greater detail in Table 5 and Table 6.

ALL demonstrated a lower reoperation rate (OR 0.7; *p* = 0.01). No difference was observed in the failure rate (*p* = 0.9). These results are shown in Table 7.

## 4. Discussion

According to the main findings of the present systematic review, LEAP for ACL reconstruction seems to be effective and safe. ACL reconstruction using LEAP led to a statistically significant improvement in PROMs and stability, with the Lysholm and the IKDC scores increasing beyond their MCID [64,65,66]. Most patients returned to their pre-injury level of sport at a mean of 6 months. The LET subgroup was associated with greater outcomes than ALL; however, the value of the IKDC did not overcome its MCID, and therefore, its clinical relevance remains uncertain. Conversely, ACL reconstruction using LET is associated with a higher reoperation rate. On the other hand, no significant differences were found between the two groups in the Lysholm score (*p* = 0.6) and Tegner score (*p* = 0.2). These results may encourage ACL reconstruction using ALL (Table 8).

Arthroscopic ACL reconstruction is the gold standard for restoring joint stability and function after an ACL tear [102,103]. LEAP has emerged as an additional procedure to enhance rotational stability following ACL reconstruction, improving general outcomes and lowering the risk of failure [104,105,106]. LET and ALL ACL reconstructions are examples of LEAP that improve rotational stability [29,31]. LET reinforces the lateral side of the knee and usually involves fixing a part of the ITB to the femur above the knee joint. In contrast, ALL consists of a more anatomical reconstruction of the anterolateral ligament with a free graft between the tibia and the femur [107,108]. LET and ALL ACL reconstructions vary mainly in surgical execution and anatomical targets [107,109,110,111]. There has yet to be a consensus on the ideal surgical procedure, and numerous studies have evaluated these techniques over the past years, focusing on PROMs, knee laxity, return to sport, and safety [31,32,33].

Previous studies evaluating LEAP for ACL reconstruction have shown controversial results. Coquard et al. [76], comparing 222 patients treated with traditional ACL reconstruction versus ALL ACL reconstruction, also reported no significant difference in the Tegner score. Bo-Ram et al. [33] conducted a meta-analysis including 20 studies involving 2376 patients comparing ACL in isolation versus ACL combined with LEAPs (LET or ALL). Despite some limitations related to the heterogeneity of the reported studies and the subjectivity in measurement, ACL reconstruction using LEAPs showed slight improvements in subjective scores [33]. LET and ALL reduced instability, but ALL performed slightly better, given its more anatomic approach [33]. ALL, in particular, significantly improves rotational stability and graft failure reduction when combined with ACL reconstruction [33]. Boksh et al. [112] recently conducted a systematic review evaluating ten comparative clinical studies (793 patients): 390 patients underwent isolated ACL reconstruction, and 403 received ACL reconstruction with augmentation (ALL or LET). Augmentation significantly improved IKDC scores compared to isolated ACL reconstruction and demonstrated better rotational stability [112]. The graft failure rate in this cohort was considerably lower, and few complications were reported [112]. Ho Jong Ra et al. [113] conducted a systematic review of 16 studies with 1442 patients, subdivided into 1048 receiving ACL reconstruction and ALL and 394 receiving ACL reconstruction using LET. Both techniques yield similar patient-reported functional outcomes, but ALL provides superior anterior knee stability compared to LET, with fewer complications [113]. While effective for rotational stability, LET has limitations due to overconstraint, non-anatomic graft orientation, and tension variability [113].

In the present study, joint laxity after LEAP was evaluated clinically using arthrometry, the Pivot Shift, and Lachman tests. A few patients still exhibited residual laxity, as assessed through the physical examination. Ibrahim et al. [83] found less anterior translation in the ALL group than in traditional reconstruction in 110 patients. Both procedures (ALL and LET) improve rotational stability [33]. ALL reconstruction may lead to better rotational stability, a lower risk of stiffness, and a lower rate of complications compared to LET [33]. Addressing rotational instability is crucial for decreasing secondary soft tissue damage risk [114]. LET seems to be associated with a higher risk of stiffness. A systematic review of 20 studies reported that knee stiffness was present in 10 studies (1284 patients), with a loss of full extension or flexion of >5° [33]. The variability in individuating instability and knee stiffness among different studies may be attributed to the varying definitions of stiffness and stability used by other authors [33]. In detail, a higher rate of patients in the ALL group was positive for the Lachman test, suggesting a higher residual anterior knee laxity compared to the LET group [33]. However, no differences were found between ALL and LET when tested with the KT-1000 arthrometer and the Pivot Shift test. A negative Lachman test has been described in most patients treated with LET [80]. On the other hand, LET combined with ACL reconstruction had significantly worse anterior knee stability than ALL in ACL reconstruction, with a higher proportion of knees graded as 2 or 3 on the Lachman test of 10.8% in the LET and 1.5% in the ALL [113]. The large difference in these findings across studies can be attributed to the low accuracy of the Lachman test [115].

In the present investigation, 72.3% of patients returned to sport within an average of 6.3 months after surgery. This finding emphasises the potential of LEAP ACL reconstruction in restoring knee function and enabling athletes to return to their previous competitive levels. A retrospective single-centre investigation comparing traditional versus ALL ACL reconstruction reported similar results in return to sport at the same level and return to competitive sport [75]. A greater rate of return to sport was seen in the LET group compared to the ALL group [75]. However, no significant differences were observed regarding the time to return to sport [75]. This finding regarding LET must be balanced with the associated higher reoperation rate observed in this cohort. The reason for reoperation is related to several complications [91]. LET can lead to overconstraint, particularly with techniques that involve rigid fixation or improper tensioning, which may cause limited knee motion and an increased risk of joint degeneration [116,117,118]. Furthermore, techniques using staples or other fixation devices have been associated with hardware irritation and subsequent hardware removal [81]. Some patients undergoing combined procedures required secondary meniscal procedures, such as meniscectomies or repairs, but these were not directly linked to LET [119]. Infections, hemarthrosis, cyclops lesions, and fibrous nodules are other causes of reoperation [120]. Marshall et al. [120] have reported that the most frequent complications associated with LET are graft failure, hematoma, infection, chronic pain, tunnel convergence, fixation device migration, muscular hernia, peroneal nerve palsy, and knee stiffness. The most frequent complications that do not influence the reoperation rate are anterior knee pain, symptomatic tibial tunnel cyst, dysesthesia, hemarthrosis, and growth disturbance [91]. While LET may provide higher early functional improvements, it is also associated with a higher risk of reoperation owing to the more invasive nature of the procedure. Indeed, LET usually requires extensive soft tissue dissection, potentially leading to complications such as stiffness, pain, or surgical site infections that may require reoperation [33,121,122].

According to the present systematic review, LET is associated with a slightly earlier functional recovery. Therefore, it may be suitable for specific cases needing a quick return to functional activities. Complications, including graft failure and reoperation, must be considered when deciding on the best surgical treatment [33]. The reoperation rate due to graft rupture is lower with LEAP than with isolated ACL reconstruction [75,104,123,124]. Pettinari et al. [104] conducted a retrospective, nonrandomised, matched-pair comparative analysis on 1102 patients over the age of 30. The LEAP group had a significantly lower graft failure rate—0.7% in the LEAP group versus 2.7% in the isolated ACL reconstruction group [104]. The authors also reported that patients aged 30-35 had a significantly higher risk of graft failure than those aged over 35 years old [104]. Furthermore, the LEAP group had a significantly lower rate of secondary meniscectomy, 2.2% versus 5.6%, compared to the isolated ACL reconstruction group [104]. Given its lower costs and comparable clinical outcomes, ACL reconstruction using LET is more cost-effective than ALL [125]. The lower incidence of graft failure in augmented ACL reconstruction can be explained by the shared spreading of load, which reduces micromovement of the graft in the tunnel, allowing a successful bone–tendon healing interface and a satisfactory return to sport [96,126,127]. In the present investigation, no differences were observed in failure rates between the LET and ALL groups. Both groups evidenced similar complication rates. This finding validates the overall safety profile of both procedures. Nevertheless, although not statistically significant, LET evidenced a greater rate of reoperations (9.6% versus 13.8%). Additional investigations are required to establish possible differences in the long term. Guzzini et al. [80], in a case series of 16 elite female football players treated with LET ACL reconstruction, reported no complications or re-ruptures, showing excellent outcomes in terms of stability, functional recovery, and return to sport. Similar results were found by Heard et al. [81], who reported that LET ACL reconstruction leads to a significantly lower graft rupture than traditional ACL reconstruction. Mechanisms behind the reported differences between LET and ALL are not fully understood. Some authors have conducted a biomechanical comparison of these techniques using cadaveric specimens, showing no data supporting the advantage of one over the other but indicating that both procedures led to optimal biomechanical results [128].

Several limitations are evident. The heterogeneity of the included studies in terms of follow-up length, variability in demographics, and differences in surgical techniques and rehabilitation protocols may reduce the validity of the present study’s results. Furthermore, this study mainly focused on clinical results and PROMs without evaluating any other diagnostic tests or imaging techniques, such as MRI, to assess the healing of the ligament. Only studies with a minimum follow-up of six months were considered. Arthroscopic management of ACL tears involves structural modifications to the knee joint, and the recovery process and the durability of outcomes often extend beyond the early postoperative period. Studies with a follow-up period shorter than 24 months may fail to capture late complications, secondary procedures, or the full extent of functional recovery, particularly in active populations like athletes. In addition, a minimum follow-up of 24 months is widely accepted in orthopaedic and sports medicine research as a standard for evaluating mid- to long-term outcomes. This timeframe ensures that the reported results reflect stable clinical and functional outcomes rather than transient or incomplete recoveries.

Most studies lacked a control group. Given the lack of quantitative data on the endpoints of interest, a meta-analysis was not possible. Given the lack of quantitative data and missing information, the time from injury to surgery was not analysed separately. Ferretti et al. [78] reported that the Tegner score improves significantly in LEAP ACL reconstruction in 100 patients, but a lower Tegner score is observed in patients with chronic tears. Another study [82] found no differences in knee stability, PROMs, complications, or failures in 130 acute versus chronic ALL ACL reconstructions, showing promising results regardless of the timing of surgery after injury. The lack of long-term data, particularly regarding graft longevity and long-term joint health, remains an essential gap in the literature [76]. While valuable, the reliance on PROMs and clinical tests may not fully capture the results of LEAP in facilitating return to sport, preventing new injuries, or mitigating degenerative changes. Furthermore, other vital tests reported in only a few studies that can give important information should be examined, including single-leg hop distance (SLHD), Limb Symmetry Index (LSI), and the Tampa Scale for Kinesiophobia (TSK). More studies are needed to compare these two procedures and explore their relative risks and advantages by establishing standardised criteria with defined outcomes. Future high-quality comparative studies with longer follow-up periods and RCTs are needed to validate the present results in a clinical setting.

## 5. Conclusions

LEAP for ACL reconstructions seems to be effective and safe. This procedure restores rotational stability, leading to good clinical results, optimal functional outcomes, and great patient satisfaction. Most patients returned to their pre-injury level of sport at a mean of 6 months. LET ACL reconstruction may be associated with greater clinical outcomes, but, on the other hand, it could be associated with a higher reoperation rate compared to ALL reconstruction.

## Figures and Tables

**Table 1:** Characteristics and patient baseline of the included studies.

Author and Year	Journal	Procedure	Follow-Up (Months)	Patients (*n*)	Women (*n*)	Mean Age (Years)	Mean BMI
Arnaud Gonnachon et al., 2024 [75]	*Eur. J. Orthop. Surg. Traumatol.*	ALL	54	41	8	24.0	
Coquard et al., 2022 [76]	*Arthrosc. Sports Med. Rehabil.*	ALL	6	111	47	37.7	24.6
El-Azab H et al., 2023 [77]	*Injury*	LET	24	50	8	28.0	
Ferretti et al., 2009 [78]	*Knee Surg. Sports Traumatol. Arthrosc.*	LET	72	100	23	35.0	
Ferretti et al., 2022 [79]	*Am. J. Sports Med.*	LET	188	79	14	26.1	
Guzzini et al., 2016 [80]	*Int. Orthop.*	LET	73	18	16	24.9	
Heard et al., 2023 [81]	*J. Isakos*	LET	24	306	155	19.1	24.0
Helito CP et al., 2023 [82]	*Arthroscopy*	ALL	29	34		27.2	
		ALL	29	96		29.6	
Ibrahim et al., 2017 [83]	*Am. J. Sports Med.*	ALL	27	56	0	26.0	
Joseph et al., 2020 [84]	*J. Exp. Orthop.*	LET	96	35	7	23.0	23.5
Laboudie et al., 2022 [85]	*Knee Surg. Sports Traumatol. Arthrosc.*	ALL	36	102	40	16.3	22.1
Lee et al., 2023 [86]	*Orthop. J. Sports Med.*	ALL	29	39		30.4	19.7
Lee et al., 2024 [87]	*Sci. Rep.*	LET	12	24	11	29.4	25.8
Legnani et al., 2022 [88]	*J. Comp. Eff. Res.*	LET	74	16	5	26.8	22.9
Marcacci et al., 2009 [89]	*Am. J. Sports Med.*	LET	132	54	12		
Meynard et al., 2020 [90]	*Orthop. Traumatol. Surg. Res.*	LET	120	50	17	28.5	25.4
Monaco et al., 2022 [91]	*Am. J. Sports Med.*	LET	44	71	27	16.1	
Pioger et al., 2022 [92]	*Am. J. Sports Med.*	ALL	101	1009		25.8	
Porter et al., 2022 [93]	*ANZ J. Surg.*	LET	24	80	42	23.0	21.3
Saragaglia et al., 2013 [94]	*Int. Orthop.*	LET	76	68	22	29.7	
Sonnery-Cottet et al., 2015 [95]	*Am. J. Sports Med.*	ALL	32	92	24	24.0	
Sonnery-Cottet et al., 2017 [96]	*Am. J. Sports Med.*	ALL	35	221	69	21.8	
Thaunat et al., 2017 [97]	*Am. J. Sports Med.*	ALL	36	548	163	24.3	
Vadalà et al., 2013 [98]	*Int. Orthop.*	LET	45	28	28	26.0	
Yang et al., 2023 [99]	*Arthroscopy*	ALL	48	35	7	25.0	26.6
Zaffagnini et al., 2006 [100]	*Knee Surg. Sports Traumatol. Arthrosc.*	ALL	60	25	7	26.7	
Zaffagnini et al., 2008 [101]	*Scand. J. Med. Sci. Sports*	ALL	46	35	15	26.0	25.1

**Table 2:** Baseline comparability.

Endpoint	ALL (N = 2444)	LET (N = 979)	*p*
Women	31.4% (380 of 1210)	39.5% (387 of 979)	0.6
Mean follow-up (weeks)	61.5 ± 34.2	62.7 ± 50.4	0.1
Mean age	25.4 ± 3.6	24.2 ± 5.6	0.4
Mean BMI	23.5 ± 2.0	23.7 ± 1.2	0.9
Lysholm	62.2 ± 15.1	58.7 ± 12.0	0.5
IKDC	58.0 ± 18.2	58.5 ± 12.4	0.7

**Table 3:** Results of PROMs (MD: mean difference; FU: follow-up; IKDC: International Knee Documentation Committee).

PROMs	At Baseline	At Last FU	MD	*p*
Lysholm	60.3 ± 13.6	91.8 ± 10.7	31.4	<0.01
IKDC	58.2 ± 16.3	87.2 ± 10.5	29.1	<0.01

**Table 4:** Results of laxity tests, Tegner activity scale, and return to sport.

Endpoint	At Last Follow-Up
Tegner	6.8 ± 2.0
KT 1000 (mm)	1.5 ± 1.8
Pivot shift	15.8% (105 of 666)
Lachman test	13.0% (87 of 668)
Return to sport	72.3% (623 of 862)
Time to return to sport (months)	6.3 ± 4.4

**Table 5:** Results of PROMs, laxity tests, and return to sport (IKDC: International Knee Documentation Committee; MD: mean difference; OR: odds ratio).

Endpoint	ALL	LET	Measure	Effect Size	*p*
Lysholm	90.8 ± 11.3	93.5 ± 9.0	MD	2.7	0.6
IKDC	84.1 ± 12.3	89.8 ± 8.0	MD	5.6	0.04
Tegner	7.2 ± 1.9	5.3 ± 1.9	MD	−1.9	0.2
Arthrometer (mm)	1.1 ± 1.5	2.0 ± 2.1	MD	0.9	0.2
Pivot shift	13.0% (32 of 247)	17.4% (73 of 419)	OR	0.7	0.1
Lachman test	20.3% (50 of 246)	8.8% (37 of 422)	OR	3.1	<0.01

**Table 6:** Percentage and time of return to sport for both procedures.

Endpoint	ALL	LET	Measure	Effect Size	*p*
Return to sport	67.6% (375 of 555)	80.8% (248 of 307)	OR	0.5	<0.01
Time to return to sport (months)	6.4 ± 4.4	6.1 ± 0.2	MD	−0.3	0.3

**Table 7:** Results of the outcome: complications (OR: odds ratio).

Endpoint	ALL (N = 2444)	LET (N = 979)	OR	*p*
Failure	3.0% (69 of 2333)	3.2% (27 of 838)	0.9	0.7
Reoperations	9.6% (183 of 1906)	13.8% (54 of 392)	0.7	0.01

**Table 8:** Advantages and disadvantages of LET and ALL.

Feature	LET Advantages	LET Disadvantages	ALL Advantages	ALL Disadvantages
Clinical Outcomes	Higher IKDC score at last follow-up	Higher reoperations rate	Good rotational stability and lower graft failure rates	Lower IKDC score
Return to sport	Greater percentage of return to pre-injury levels	No significant difference in time to return to sport	Comparable time to return to sport	Lower percentage of return to pre-injury sport level
Laxity and Stability	Lower positive rate on Lachman test (better anterior knee stability)	Slightly higher laxity noted in arthrometer and Pivot shift tests	Superior in reducing anterior translation and residual rotational instability	Higher positive rate on Lachman test (worse anterior knee stability)
Complications	Effective for rotational stability	May cause overconstraint and stiffness. Increased risk of surgical site complications (e.g., infections and fibrosis)	Fewer complications and better anterior knee stability	Some residual anterior knee laxity observed
Reoperation Rates		Higher rates	Lower rates	Risks of surgical failure in chronic conditions

## Data Availability

Data are contained within this article.
